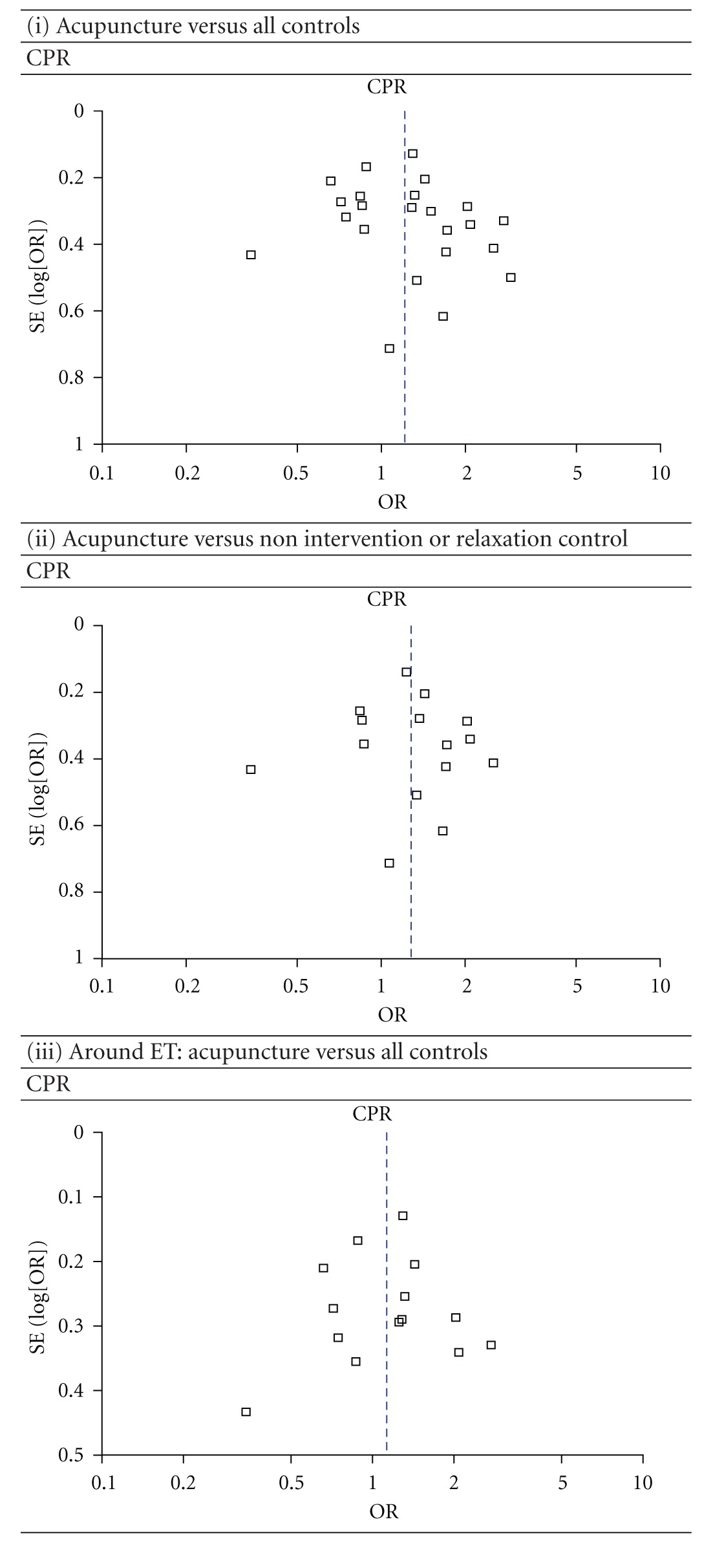# The Role of Acupuncture in Assisted Reproductive Technology

**DOI:** 10.1155/2012/543924

**Published:** 2012-07-02

**Authors:** Cui Hong Zheng, Ming Min Zhang, Guang Ying Huang, Wei Wang

**Affiliations:** ^1^Institute of Integrated Traditional Chinese and Western Medicine, Tongji Hospital, Tongji Medical College, Huazhong University of Science and Technology, Wuhan, Hubei 430030, China; ^2^Department of Integrated Traditional Chinese and Western Medicine, Tongji Hospital, Tongji Medical College, Huazhong University of Science and Technology, Wuhan, Hubei 430030, China; ^3^Department of Neurology, Tongji Hospital, Tongji Medical College, Huazhong University of Science and Technology, Wuhan, Hubei 430030, China

## Abstract

The aim of this paper was to provide reliable evidence by performing a systematic review and meta-analysis for evaluating the role of acupuncture in assisted reproductive technology. All randomized controlled trials that evaluated the effects of acupuncture, including manual, electrical, and laser acupuncture (LA) techniques, on the clinical pregnancy rate (CPR) and live birth rate (LBR) of in vitro fertilization (IVF) or artificial insemination were included. The controlled groups consisted of no acupuncture and sham acupuncture groups. The sham acupuncture included sham acupuncture at acupoints, sham acupuncture at non- or inappropriate points, sham LA, and adhesive tapes. Twenty-three trials (a total of 5598 participants) were included in this paper. The pooled CPR from all acupuncture groups was significantly higher than that from all controlled groups, whereas the LBR was not significantly different between the two groups. However, the results were quite distinct when the type of control and/or different acupuncture times were examined in a sensitivity analysis. The results mainly indicate that acupuncture, especially around the time of the controlled ovarian hyperstimulation, improves pregnancy outcomes in women undergoing IVF. More positive effects from acupuncture in IVF can be expected if a more individualized acupuncture programs are used.

## 1. Introduction

Acupuncture is an important part of traditional Chinese medicine (TCM) that dates back at least 3000 years. Acupuncture can cure disease because it can stimulate the body's self-regulatory ability that is characterized by integrity and ambidirectional dominance. Acupuncture has gained increased popularity in western countries due to its convenience, lack of side effects, and unique therapeutic effects [1]. As a method of treating disease, acupuncture is based on the principles of TCM meridians and acupoints. Meridians are the main and collateral channels of a network of passages through which vital energy circulates and along which acupoints are distributed. There are 14 main meridians, upon which more than 300 acupoints are located. Acupoints are not isolated, they are special points on the surface of the body where the vital energy (qi and blood) of the viscera infuses. In other words, there are inherent relationships between acupoints and internal organs that correspond loosely to the organs of western medicine. Therefore, diseases of the entrails may be reflected in acupoints through meridians, and acupuncture at acupoints can affect the corresponding organs through meridians. Traditional acupuncture involves inserting disposable sterilized needles into the skin at acupoints along the meridians. The needles can then be stimulated by hand or by a small electric current in the case of electroacupuncture (EA). Laser acupuncture (LA) is a new form of this treatment; it combines modern science and technology with traditional methods by using a low-energy laser beam to directly irradiate acupoints.

In vitro fertilization-embryo transfer (IVF-ET) is the most successful infertility treatment, and for many people, it provides the last possibility for pregnancy. However, the average IVF delivery rate per single initiated cycle using fresh, nondonor oocytes is still only 33% [2]. The majority of IVF cycles do not result in pregnancy. Due to the relatively low IVF success rate per cycle, some patients are not successful even after several ETs, even when the appropriate techniques for controlled ovarian hyperstimulation (COH), in vitro fertilization, embryo culture and transfer, and so forth are correctly performed. Similarly, the success rate of intrauterine insemination (IUI) is also not satisfactory. Therefore, repeated cycles will place enormous economic pressure on the patients and their families.

Since the first report by Stener-Victorin et al. [3] in 1999 suggesting that acupuncture can increase the clinical pregnancy rate (CPR) of IVF, the application of acupuncture to assisted reproductive technology (ART) has attracted great interest from the international community. More than 40 clinical trials evaluating acupuncture in IVF have been performed in recent years. However, whether acupuncture improves IVF pregnancy rates is still a matter of debate. Some studies have suggested a positive impact from adding acupuncture to IVF, but there are others that do not confirm this effect. Seven systematic reviews and meta-analyses of randomized controlled trials (RCTs) have investigated the ability of acupuncture to increase IVF success rates. However, these meta-analyses have led to contradictory conclusions.

The first meta-analysis was performed by Manheimer et al. (7 trials with 1,366 participants) and was published in the BMJ in February 2008 [4]. The main conclusions of this study were that acupuncture given around ET improved the rates of clinical pregnancy, ongoing pregnancy, and live birth in women undergoing in vitro fertilization. The second analysis was conducted by Ng et al. (10 trials with 2,003 subjects) and published in Fertility and Sterility in July 2008 [5]; it clearly demonstrated that the IVF pregnancy rate is significantly increased, especially when the acupuncture is administered on the day of embryo transfer. The third analysis, published by Cheong et al. [6] in the Cochrane Collaboration in 2008 (13 trials, 2,300 participants), concluded that acupuncture performed on the day of the embryo transfer increases live birth rates but does not increase clinical pregnancy rates, and there are no beneficial effects on pregnancy outcomes when acupuncture was performed around the time of oocyte retrieval. The other four meta-analyses, published by El-Toukhy et al. (13 trials, 2,500 participants) [7], Cheong et al. (14 trials, 2,670 subjects) [8], El-Toukhy and Khalaf [9], and Sunkara et al. [10] (14 trials, 2,870 subjects), could not confirm a beneficial effect from using acupuncture during IVF. 

Why did these meta-analyses addressing the same question producing such different answers? Systematic reviews and meta-analyses are generally regarded as the most reliable tool for summarizing the existing evidence. However, they often show differences in their results and conclusions. The most common reasons for these discrepancies are differences in inclusion criteria, methods of searching the literature, data extraction, and data analysis [11], although all of these aspects have been considered in some way in these reviews. In particular, some older and even more recent RCTs have been ignored in these analyses. Therefore, it is difficult to draw a definitive conclusion based on the published meta-analyses. Consequently, a new comprehensive systematic review and overall meta-analysis are indispensable for drawing more reliable conclusions on the ability of acupuncture to improve pregnancy outcomes when used as an adjunct in ART.

## 2. Material and Methods

### 2.1. Search Strategy

We searched digital databases for relevant studies, including Pubmed (1977 to July 2011), EMBASE (1974 to July 2011), the Cochrane Library, and the Clinical Trials Register. We also searched Chinese databases, such as Wanfang Database (1998 to July 2011), CNKI Database (1999 to July 2011), and VIP Database (1989 to July 2011).

The following were used as free text terms and MeSH terms (shown in italics): *acupuncture; electroacupuncture; acupuncture and moxibustion; acupoint*; *IVF; in vitro fertilization; intrauterine insemination*; *artificial insemination*; *assisted conception; *and* assisted reproductive (or reproduction) technology*, and so forth. We combined this search strategy with a filter for clinical trials.

The following terms were used in the Chinese database searches: “*ZHEN JIU*” (which means “acupuncture and moxibustion”); *“ZHEN CI*” (which means “acupuncture”); “*TI WAI SHOU JING*” (which means “in vitro fertilization”); “*SHI GUAN YING ER*” (which means “test tube baby”); “*REN GONG SHOU JING*” (which means “artificial insemination”; “*GONG QIANG NEI REN GONG SHOU JING*” (which means “intrauterine insemination”; and* “FU ZHU SHENG ZHI JI SHU”* (which means* “assisted reproductive (or reproduction) technology”)*, and so forth.

We also carefully scanned the references of relevant publications and added the relevant publications to the search. When questions arose related to the design or outcomes of the trials, the corresponding authors were contacted to confirm the information we extracted from their trials or to clarify any ambiguities. 

### 2.2. Study Selection

All the RCTs that evaluated the effects of acupuncture, including manual (MA), electrical (EA), and LA techniques, on CPR or live birth rate (LBR) in women undergoing IUI or IVF with or without intracytoplasmic sperm injection (ICSI) were considered. The controlled groups consisted of no and sham intervention treatment. In principle, five styles of sham acupuncture exist: (1) superficial needling at nonacupoints nearby; (2) true needling at nonacupoints or at acupoints thought not to influence fertility; (3) blunt needling on the surface of true acupoints or nonacupoints nearby (Streitberger placebo acupuncture, e.g.); (4) electrical stimulation with no current; (5) sham LA in which the laser device indeed does not emit light pulses. It should be emphasized that neither the type, that is, full article or abstract, nor language of the publication restricted the trials included in this study.

Retrospective studies, case series, and studies with a crossover design were excluded. RCTs without a clear description of the CPR, especially those not describing the exact numbers of pregnancies (events) and initial setups (total), were also not considered.

### 2.3. Data Extraction

The literature searching, study selection, data extraction, and statistical analysis were performed independently by two reviewers (Zheng and Zhang). Any disagreements about inclusions or analyses were resolved by consensus or arbitration by a third reviewer (Huang).

Specific characteristics were extracted from each study: method of randomization, allocation concealment, blinding, sample size, population features, intervention (e.g., acupuncture style, MA, EA, or LA), time of commencement, duration of treatment, type of control (no, or sham acupuncture), number of randomizations, and pregnancy outcomes, and so forth.

The pregnancy outcomes consisted of biochemical pregnancy rate (BPR), CPR, ongoing pregnancy rate (OPR), LBR, implantation rate, miscarriage rate, and any reported side effects of treatment. The CPR is more accurate than BPR. It is difficult to obtain all the data on OPR and LBR from these included trials; however, LBR is the most significant outcome, therefore, CPR and LBR are the best to represent the true combined effect from these trials rather than the other outcomes.

### 2.4. Statistical Analysis

The pregnancy outcomes reported in these trials were pooled and expressed as odds ratios (ORs) with 95% confidence intervals (CIs) in the Review Manager 5.1 meta-analysis software. In this paper, the type of control and acupuncture time was used for sensitivity subgroup analyses. We used a fixed effects model for these meta-analyses if the heterogeneity for the trials' characteristics showed *P* > 0.05; otherwise, we used a random effects model. All the meta-analyses were based on the number of women randomized. That is, we performed an intention to treat (ITT) analysis. The heterogeneity of the therapeutic effects was evaluated graphically using a forest plot analysis and statistically using the Chi-squared test. The publication bias was assessed by funnel plots. Publication bias may lead to asymmetrical funnel plots; the asymmetry of the funnel plot was further explored by a weighted linear regression analysis (R2.0 software).

## 3. Results

### 3.1. Search Results

**Figure 1:**
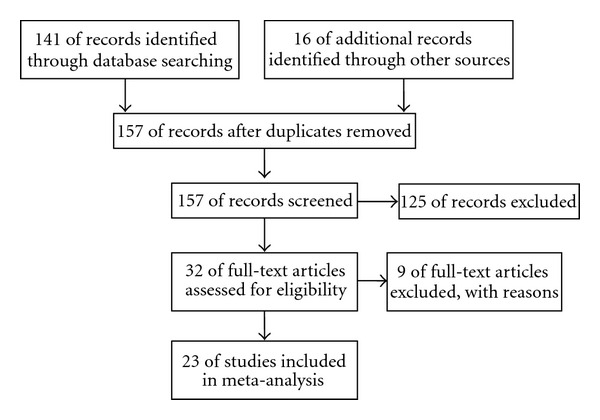
Flow diagram of study inclusion.

After identification and screening (Figure 1), thirty-two trials involving acupuncture and IVF were assessed for eligibility. Twenty-three trials (a total of 5598 participants, Table 1) were included in this review and nine were excluded.

The nine trials excluded were: Quintero [12], Udoff et al. [13], Humaidan et al. [14], Moy et al. [15], Kong and Hughes [16], Li et al. [17], Omodei et al. [18], Gejervall et al. [19], and Magarelli et al. [20]. Although the study conducted by Quintero [12] was a randomized controlled and double-blinded trial, it was also a crossover pilot study using a needle-like device for the sham acupuncture controlled. Furthermore, data for the exact pregnancy events and totals were not available because the trial only used PR, which was also the reason for excluding Udoff et al. 2006 [13], Moy et al. 2008 [15], and Omodei et al. 2010 [18]. Both Humaidan et al. 2006 [14] and Kong et al. 2009 [16] were RCTs, but the control was a real acupuncture group with only the stimulation parameter differing from the intervention group. The data on the number of cancelled IVF cycles in Li et al. 2009 [17] was inconsistent; therefore, we excluded it. The study conducted by Gejervall et al. 2005 [19] was excluded because there was only BPR data, not CPR or LBR although it was a RCT. Magarelli et al. 2009 [20] was not a randomized trial.

### 3.2. Characteristics of Studies

#### 3.2.1. Publishing Form

Eighteen trials were published as full text, and five [22, 26, 27, 29, 37] were published as abstracts. Twenty trials were published in English and three [28, 30, 39] in Chinese.

#### 3.2.2. Country

The trials were conducted in nine different countries. Three of them were performed in fertility clinics in Germany [21, 22, 25], five were from the United States [26, 27, 29, 31, 38], and one each was from Australia [23], Brazil [36], Italy [37], and Austria [42]. Two studies were performed in Sweden [3, 40], six were from China [28, 30, 32–34, 39], and three were from Denmark [24, 35, 41].

#### 3.2.3. Centers

Four studies [3, 27, 35, 40] were multicenter trials, while the remaining 19 were performed in a single centre.

#### 3.2.4. Objectives and Outcomes

 Four [3, 40–42] of these trials were performed to evaluate the pain-relieving effects of acupuncture used around the time of oocyte aspiration (OA), and two [41, 42] of these four studies calculated the required sample size according to the primary objective rather than the secondary IVF outcome. The remaining 19 trials were designed to assess the effects of acupuncture on pregnancy rates from IVF, but only ten of them used a sample size sufficient to detect an effect on IVF outcomes between the study groups. Twelve trials performed ITT analysis, eight performed TPP analysis, and three performed both ITT and TPP (Table 1).

#### 3.2.5. Interventions and Controls

As shown in Table 1, 16 trials used MA as an adjunctive treatment, two of which also utilized LA as a second intervention group [7, 10], and Westergaard et al. [5] used two MA intervention groups and one controlled group. Eight studies used EA.

Five studies used Streitberger acupuncture as control: Smith et al. [23] used this sham acupuncture at points close to the real points, and Paulus et al. [22], So et al. [33, 34], and Andersen et al. [35] used the sham acupuncture in a manner identical to the acupuncture used in the study group. Dieterle et al. [25] used an actual needling procedure at acupoints that were designed not to affect fertility, Benson et al. [26] and Fratterelli et al. [29] used sham LA, Sator-Katzenschlager et al. [42] used adhesive tape instead of needles and no electrical stimulation, and Moy et al. [38] used needles at nonacupoints. Fifteen studies used non intervention or relaxation as the controlled group. Both Benson et al. and Fratterelli et al. had two intervention groups (MA and LA) and three controlled groups (sham LA, relaxation, and non intervention).

As for the statistical analyses, we classified all of the controls into five categories: sham acupuncture at acupoints, sham acupuncture at non- or inappropriate points, non intervention or relaxation controlled, sham LA, and adhesive tapes.

#### 3.2.6. Acupuncture Time

We divided the trials into three types according to their acupuncture times (Table 1). In type A, the acupuncture was performed around the time of the ET. An example of type A is the study by Paulus et al. [21], which performed two 25-minute sessions immediately before and after the ET. In type B, the acupuncture was performed around the time of the OA. An example of type B is the study by Stener-Victorin et al. [3], which began at least 30 min before the OA and terminated directly after the OA. In type C, the acupuncture was mainly performed during the course of the COH, and four or more sessions were administered. An example of type C is the study by Ho et al. [32], which administered treatments four times, twice a week for 2 weeks, from day 2 of the study to the day before the OA. There were a total of 14 type A trials, 4 type B trials, and 5 type C trials (Table 1).

### 3.3. Effect Sizes

#### 3.3.1. Compared by Types of Control (Table 2)

**3.3.1.1. Comparison with All Controlled Groups**

The CPR data were available from 23 trials. There was significant heterogeneity between these trials (*P* = 0.0003 for the heterogeneity test). Using the random effects model, the pooled result showed a clear significant difference between all acupuncture groups and all controlled groups (*n* = 5598, 39.5% versus 37.2%, *P* = 0.05, OR = 1.21, 95% CI [1.00 to 1.46]). The LBR data were available from 6 trials. The pooled result was not significantly different between the two groups (*n* = 2396, 32.8% versus 31.6%, *P* = 0.86, OR = 1.03, 95% CI [0.76 to 1.40]).

**3.3.1.2. Comparison with Sham Acupuncture at Acupoints**

The CPR data were available from 4 trials. There was no significant heterogeneity between these trials (*P* = 0.27 for the heterogeneity test). Using the fixed effects model, the pooled result showed no significant difference between the acupuncture groups and the sham acupuncture at acupoints groups (*n* = 1431, 36.1% versus 40.3%, *P* = 0.09, OR = 0.83, 95% CI [0.67, 1.03]). The pooled LBR from the acupuncture groups was significantly lower than that from the sham acupuncture groups (3 studies, *n* = 1231, 27.3% versus 33.4%, *P* = 0.02, OR = 0.74, 95% CI [0.58, 0.95]).

**3.3.1.3. Comparison with Sham Acupuncture at Non- or Inappropriate Points**

The pooled CPR showed no significant difference between the acupuncture groups and the sham acupuncture at non- or inappropriate points groups (3 studies, *n* = 613, 35.9% versus 27.6%, *P* = 0.31, OR = 1.45, 95% CI [0.70, 2.98]).

**3.3.1.4. Comparison with Non Intervention or Relaxation Controlled**

The pooled CPR and LBR from the acupuncture groups were, respectively, significantly higher than those from the *non intervention or relaxation* controlled groups (CPR, 15 studies, *n* = 3210, 41.4% versus 36.7%, *P* = 0.03, OR = 1.27, 95% CI [1.03, 1.58]; LBR, 3 studies, *n* = 1165, 37.7% versus 29.2%, *P* = 0.01, OR = 1.38, 95% CI [1.07, 1.77]).

**3.3.1.5. Comparison with Sham LA**

The pooled CPR result from the acupuncture groups was significantly higher than that from the sham LA groups (2 studies, *n* = 1011, 52.6% versus 44.5%, *P* = 0.01, OR = 1.38, 95% CI [1.08 to 1.77]). The LBR showed no significant difference between the two groups (1 studies, *n* = 600, 40.5% versus 35.4%, *P* = 0.22, OR = 1.25, 95% CI [0.88 to 1.77].

**3.3.1.6. Comparison with Adhesive Tapes**

The pooled CPR from the acupuncture groups was significantly higher than that from the adhesive tapes controlled groups (1 studies, *n* = 94, 46.9% versus 23.3%, *P* = 0.03, OR = 2.90, 95% CI [1.09 to 7.71]).

#### 3.3.2. Compared by Different Acupuncture Times and Controls (Table 3)

**3.3.2.1. Around the Time of ET**

The pooled CPR and LBR results from the studies in which acupuncture was performed around the time of the ET showed no significant differences between all acupuncture groups and all controlled groups (CPR: 14 studies, *n* = 4418, 40.5% versus 39.0%, *P* = 0.32, OR = 1.12, 95% CI [0.89, 1.42]; LBR: 5 studies, *n* = 2647, 32.7% versus 34.2%, *P* = 0.67, OR = 0.97, 95% CI [0.82, 1.14]). The results of “around ET: acupuncture versus sham acupuncture at acupoints” and “around ET: acupuncture versus sham acupuncture at non- or inappropriate acupoints” were, respectively, identical to that in “*3.3.1.2” and “3.3.1.3”. *The results of* “*around ET: acupuncture versus non intervention or relaxation control” showed no significant differences between the two groups (CPR: 7 studies, *n* = 2374, 44.6% versus 40.7%, *P* = 0.13, OR = 1.27, 95% CI [0.93, 1.72]; LBR, 2 studies, *n* = 1416, 38.2% versus 34.9%, *P* = 0.12, OR = 1.19, 95% CI [0.96, 1.49]).

**3.3.2.2. Around the Time of OA**

The pooled CPR and LBR results from the studies in which acupuncture was performed around the time of the OA showed no significant differences between all acupuncture groups and all controlled groups (CPR: 4 studies, *n* = 717, 39.2% versus 36.5%, *P* = 0.48, OR = 1.12, 95% CI [0.82. 1.52]; LBR: 1 studies, *n* = 142, 33.3% versus 19.4%, *P* = 0.06, OR = 2.08, 95% CI [0.96, 4.50]). The results were a little different when the type of control was examined in a subgroup analysis. Acupuncture versus non intervention or relaxation controlled: CPR, 3 studies, *n* = 623, 37.6% versus 37.8%, *P* = 0.96, OR = 0.99, 95% CI [0.71, 1.37]; LBR, 1 studies, *n* = 142, 33.3% versus 19.4%, *P* = 0.06, OR = 2.08, 95% CI [0.96, 4.50]. Acupuncture versus adhesive tapes: 1 studies, *n* = 94, 46.9% versus 23.3%, *P* = 0.03, OR = 2.90, 95% CI [1.09 to 7.71].

**3.3.2.3. During the Time of COH**

The pooled CPR result around the time of the COH from all acupuncture groups was significantly higher than that from all controls (5 studies, *n* = 463, 31.5% versus 21.2%, *P* = 0.01, OR = 1.75, 95% CI [1.13, 2.71]). This was also the result of subgroup analysis “around COH, acupuncture versus non intervention or relaxation control”.

### 3.4. Side Effects

None of the 23 trials reported evidence of ovarian hyperstimulation or of any treatment side effects.

## 4. Discussion

### 4.1. Quality of Studies and Outcome

Although all 23 of the studies were RCTs, few provided detailed information on the randomization procedure, allocation concealment, blinding of assessors, and so forth. There was also significant clinical heterogeneity among the studies, which may have been attributable to variations in the acupuncture techniques (MA, EA, or LA), time of commencement, total dose of the intervention, method of control, acupoints, and patient populations across these studies.

Due to the nature of acupuncture studies, absolute double blinding was often not possible. Some studies that used sham acupuncture for the controlled group came near to double blinding, while others that used non intervention as the controlled were completely unblinded trials.

The regression analysis showed that there were no significant publication biases for all of the comparisons (all *P* > 0.05). The most informative funnel plots (included trials' number > 10) were shown in Table 4. 

### 4.2. Summary of Results

 In general, the quantity of trials included in this paper was substantially higher than the quantity of those included in earlier reviews. The new studies came from 3 sources: (a) Chinese databases, which were not used before; (b) studies published after the previous reviews were done; (c) a few that were ignored from previous reviews. Compared with the earlier reviews ([8], e.g.), we added 9 studies; 2 had positive results [36, 37] and 7 had negative results (there was no significant CPR or LBR difference between the acupuncture group and the controlled group: [28–30, 32, 34, 35, 38, 39]). Although many negative-result trials were added, the result of the meta-analysis showed that the pooled CPR from all of the acupuncture groups was significantly higher than that from all of the controlled groups. The results were quite distinct when the type of control was examined in a sensitivity analysis (acupuncture versus sham acupuncture at acupoints: CPR, 36.1% versus 40.3%, *P* = 0.09; LBR, 27.3% versus 33.4%, *P* = 0.02. Acupuncture versus sham acupuncture at non- or inappropriate points: CPR, 35.9% versus 27.6%, *P* = 0.31. Acupuncture versus non intervention or relaxation controlled: CPR, 41.4% versus 36.7%, *P* = 0.03; LBR, 37.7% versus 29.2%, *P* = 0.01. Acupuncture versus sham LA: CPR, 52.6% versus 44.5%, *P* = 0.01; LBR, 40.5% versus 35.4%, *P* = 0.22. Acupuncture versus adhesive tapes: CPR, 46.9% versus 23.3%, *P* = 0.03).

 The results of “acupuncture versus all controls”, “acupuncture versus non intervention or relaxation control”, “acupuncture versus sham LA”, and “acupuncture versus adhesive tapes” indicated that acupuncture really conduces to increasing the CPR and LBR, which is not psychological or placebo effect. The CPR results of “acupuncture versus sham acupuncture at acupoints” and “acupuncture versus sham acupuncture at non- or inappropriate points” showed that there were no significant differences between the acupuncture groups and sham acupuncture groups. This indicated acupuncture can induce nonspecificity effect, not only at acupoint but also at nonacupoints. However, the study by Dieterle et al. [25] signified that acupuncture at inappropriate acupoints has adverse effect on the pregnancy rate indicating that acupuncture at acupoints has some specific effect. Each acupoint has a domain. If a nonacupoint is too close to an acupoint, maybe there is no significant difference between the two effects. There are so many meridians and acupoints, known and unknown on the body so it may be not easy to define a real nonacupoint.

Why did the acupuncture group have lower LBR odds than the “sham acupuncute at acupoints” group? The “sham acupuncture at acupoints” all were Streitberger placebo controlled. The Streitberger needle is not fixed inside the copper handle. Its tip is blunt, and a pricking sensation, simulating the puncturing of the skin, is felt by the patient when it touches the skin. The needle moves inside the handle and appears to be shortened. Some researchers thought this noninvasive placebo acupuncture was the best control for acupuncture studies. However, more and more studies indicated that this placebo approach may not be an inert control. Because the patient can not feel the pricking sensation if the placebo technique is too mild, however, the acupressure effect [33] cannot be eliminated when the pressure is too heavy. Therefore, the noninvasive placebo needle used at acupoints may have elicited physiological effects similar to those of acupressure. On the other hand, the minimally invasive stimulation of acupuncture is often accompanied by some degree of discomfort or pain, which may have induced a harmful response. Therefore, the possible harmful reaction produced by real acupuncture can be avoided by this noninvasive stimulation. Therefore, the Streitberger controlled group may have had higher LBR. So, from this result we can also infer that surface stimulation at acupoints, such as acupressure or transcutaneous electrostimulation, should be considered as the adjunctive treatment in ART. It is likely that better therapeutic effects can be achieved in this manner.

When different acupuncture times were examined in a sensitivity analysis, the pooled CPR and LBR results around the time of the ET or OA showed no significant differences between all acupuncture groups and all controlled groups. However, the CPR result around the time of the COH showed significant difference between the two groups (31.5% versus 21.2%, *P* = 0.01), which means acupuncture around the time of the COH is the most suitable.

### 4.3. Study Limitations and Possible Future Improvements

 First of all, there were large heterogeneities among these clinical trials, especially in acupuncture treatment and acupoint selection. Up to the present a generally accepted standard of reference for the treatment is still missing. Both ancient and modern acupuncture books clearly emphasize that needling at some acupoints, such as Sanyinjiao, Jianjin, and Zhiyin, is not appropriate for pregnant women because an abortion may result. Therefore, using acupuncture in IVF or IUI to improve and increase the pregnancy rate expands traditional acupuncture beyond its original application range. However, different acupuncture schemes may result in different clinical effects. Even slight changes may lead to quite different clinical effects in some trials. In Craig et al. [27], for example, the acupuncture scheme was based on one reported by Paulus et al. [21], and only two acupoints were added; however, the results of the two studies were different, although the different acupuncture sites may be another influencing factor. Of course, Craig et al. was also a strange trial for it achieved a freakishly high response in the non acupuncture group (70%).

In addition, most of the courses of acupuncture treatment were too short to completely correct infertility states caused by long-term insufficiency or imbalance. Furthermore, the acupuncture programs lacked syndrome differentiation and treatment according to individual characteristics. Therefore, some experts have predicted that better therapeutic efficacy can be achieved by performing a more individualized acupuncture program [43].

Placebo controlled is commonly used in clinical trials to exclude psychological factors. However, it is difficult to establish a reasonable and suitable control in clinical acupuncture research; therefore, various acupuncture effects have been questioned. So, we strongly encourage active exploration of a reasonable and reliable acupuncture controlled method. However, if the sham is not an inert placebo but rather an active treatment that may affect the pregnancy outcome, using sham acupuncture as the control may confuse rather than clarify the interpretation of the effects of acupuncture on IVF outcomes [44]. Therefore, if the aim is only to evaluate the effectiveness of acupuncture, maybe we just require a non intervention control or a relaxation control.

## 5. Conclusions

These studies only related to the particular protocols used and most do not bear any relation to what would be considered as the best practice in TCM for treating infertility. This paper indicates that acupuncture, especially around the time of the COH, improves pregnancy outcomes in women undergoing IVF. Acupuncture effects consist of acu-specific and nonspecific effects. More positive effects from acupuncture in IVF can be expected if an appropriate control and more individualized acupuncture programs are used. However, we do not yet know what is the best acupuncture approach in IVF. Maybe, appropriate acupuncture times (around the time of COH or through the time of COH to the time of OA), enough treatment courses (at least four sessions), and syndrome differentiation and treatment according to individual characteristics should be emphasized in the acupuncture programs. We can design several different acupuncture groups in parallel for further observation to optimize the best program.

## Figures and Tables

**Table 1:** Characteristics of studies included in this paper.

Author	Main objective	Power calculation		Intervention				Control			Acupuncture juncture	CPR	LBR	Analysis
			MA	EA	LA	Sham Acu. at acupoints	Sham Acu. at non- or inappropriate points	NT or RX	Sham LA	Adhesive tapes				
(1) Paulus et al. [21]	IVF outcome	No	*√*					*√*			A	*√*		ITT
(2) Paulus et al. [22]	IVF outcome	No	*√*			*√*					A	*√*		ITT
(3) Smith et al. [23]	IVF outcome	Yes	*√*				*√*				A	*√*		ITT
(4) Westergaard et al. [24]	IVF outcome	Yes	*√* (2)					*√*			A	*√*		TPP
(5) Dieterle et al. [25]	IVF outcome	Yes	*√*				*√*				A	*√*		ITT
(6) Benson et al. [26]	IVF outcome	No	*√*		*√*			*√* (2)	*√*		A	*√*		ITT
(7) Craig et al. [27]	IVF outcome	No	*√*					*√*			A	*√*		TPP
(8) Cui et al. [28]	IVF outcome	No		*√*				*√*			C	*√*		ITT
(9) Fratterelli et al. [29]	IVF outcome	Unclear	*√*		*√*			*√* (2)	*√*		A	*√*	*√*	ITT
(10) Chen et al. [30]	IVF outcome	No		*√*				*√*			C	*√*		ITT and TPP
(11) Domar et al. [31]	IVF outcome,	No	*√*					*√*			A	*√*		ITT
(12) Ho et al. [32]	IVF outcome	No		*√*				*√*			C	*√*		ITT
(13) So et al. [33]	IVF outcome	Yes	*√*			*√*					A	*√*	*√*	ITT
(14) So et al. [34]	IVF outcome	Yes	*√*			*√*					A	*√*	*√*	ITT
(15) Andersen et al. [35]	IVF outcome	Yes	*√*			*√*					A	*√*	*√*	ITT and TPP
(16) Madaschi et al. [36]	IVF outcome	Yes	*√*					*√*			A	*√*	*√*	ITT
(17) Arnoldi et al. [37]	IVF outcome	Unclear	*√*					*√*			C	*√*		ITT and TPP
(18) Moy et al. [38]	IVF outcome	Yes	*√*				*√*				A	*√*		TPP
(19) Cui et al. [39]	IVF outcome	No		*√*				*√*			C	*√*		TPP
(20) Stener-Victorin et al. [3]	Pain relief	No		*√*				*√*			B	*√*	*√*	TPP
(21) Stener-Victorin et al. [40]	Pain relief	Yes		*√*				*√*			B	*√*		TPP
(22) Humaidan and Stener-Victorin [41]	Pain relief	Yes		*√*				*√*			B	*√*		TPP
(23) Sator-Katzenschlager et al. [42]	Pain relief	Yes	*√*	*√*						*√*	B	*√*		TPP

Total	19 IVF outcome, 4 Pain relief	11 yes	16(17)	8	2	4	3	15(17)	2	1	14A 4B 5C	23	6	15 ITT, 11 TPP

Note: MA: manual acupuncture; EA: electroacupuncture; LA: laser acupuncture; Acu.: acupuncture. NT: non intervention treatment; RX: relaxation. A: acupuncture was used around the time of embryo transfer (ET); B: acupuncture was used around the time of oocyte aspiration (OA); C: acupuncture was mainly performed during the course of controlled ovarian hyperstimulation (COH); CPR: clinical pregnancy rate; LBR: live birth rate; Westergaard 2006 MA (2): two MA groups; Benson 2006, Fratterelli 2008 NT, or RX (2): two controlled groups. ITT: intention to treat analysis; TPP: treated-per-protocol analysis.

**Table 2:** Forest plots of IVF outcomes as compared by types of control.

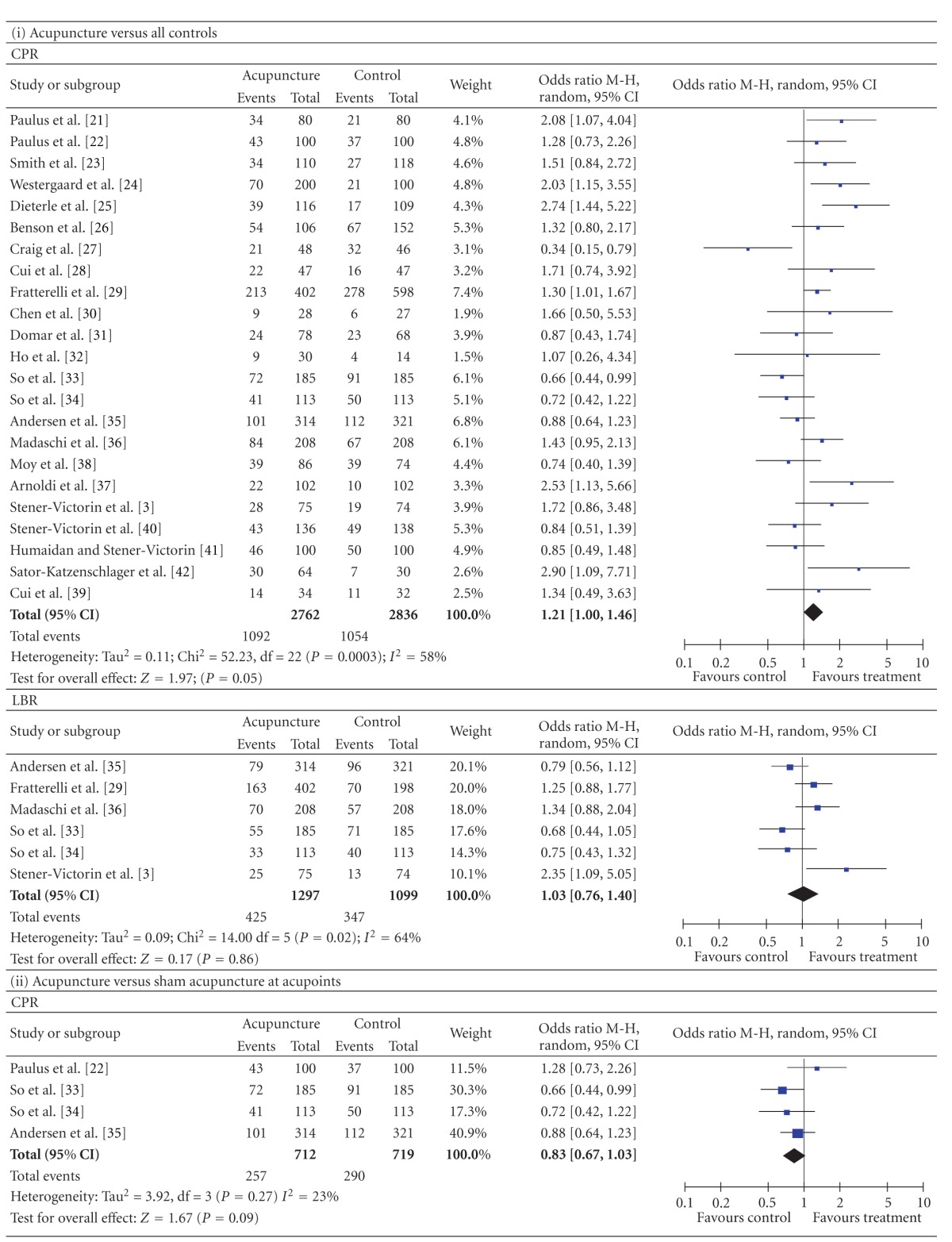 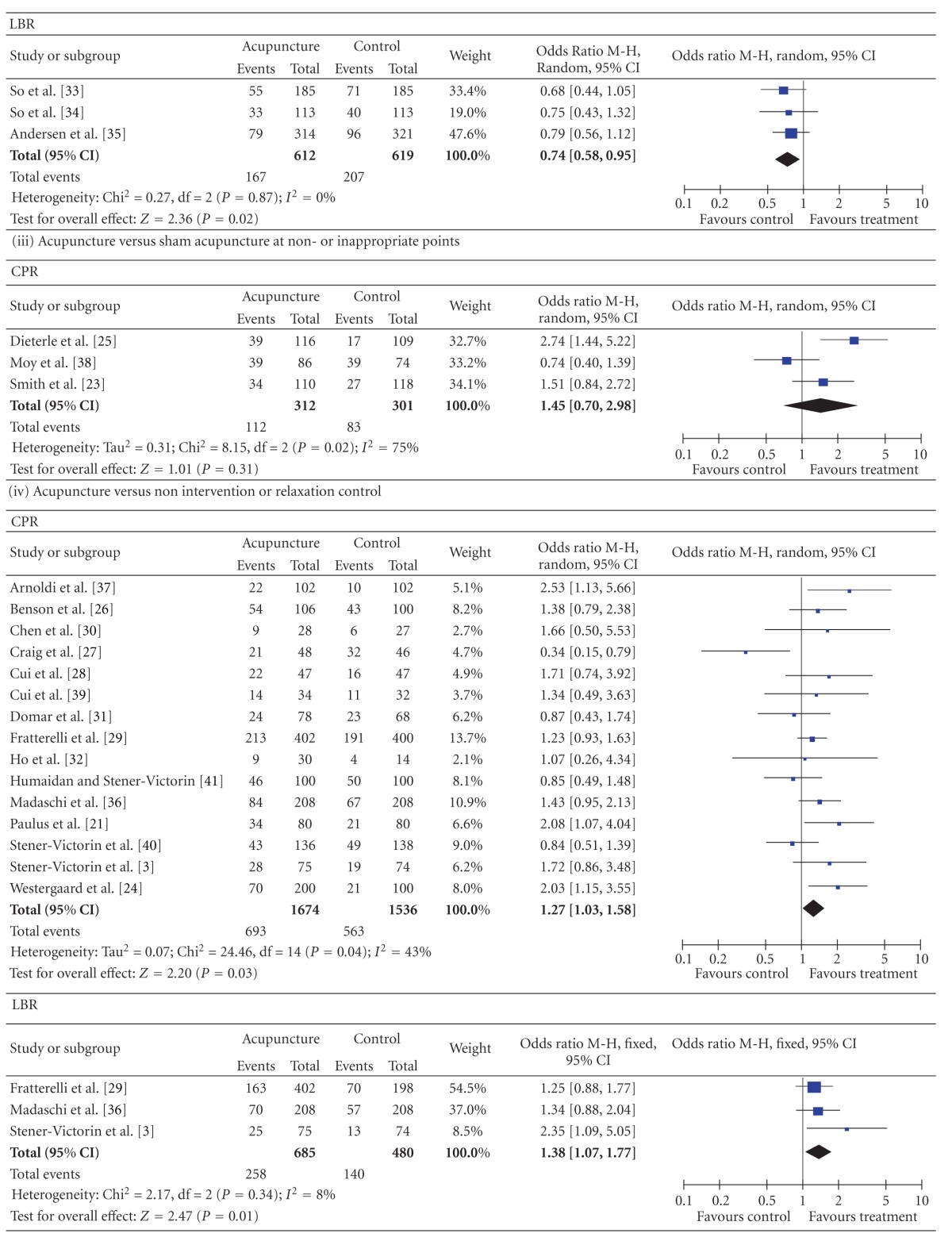 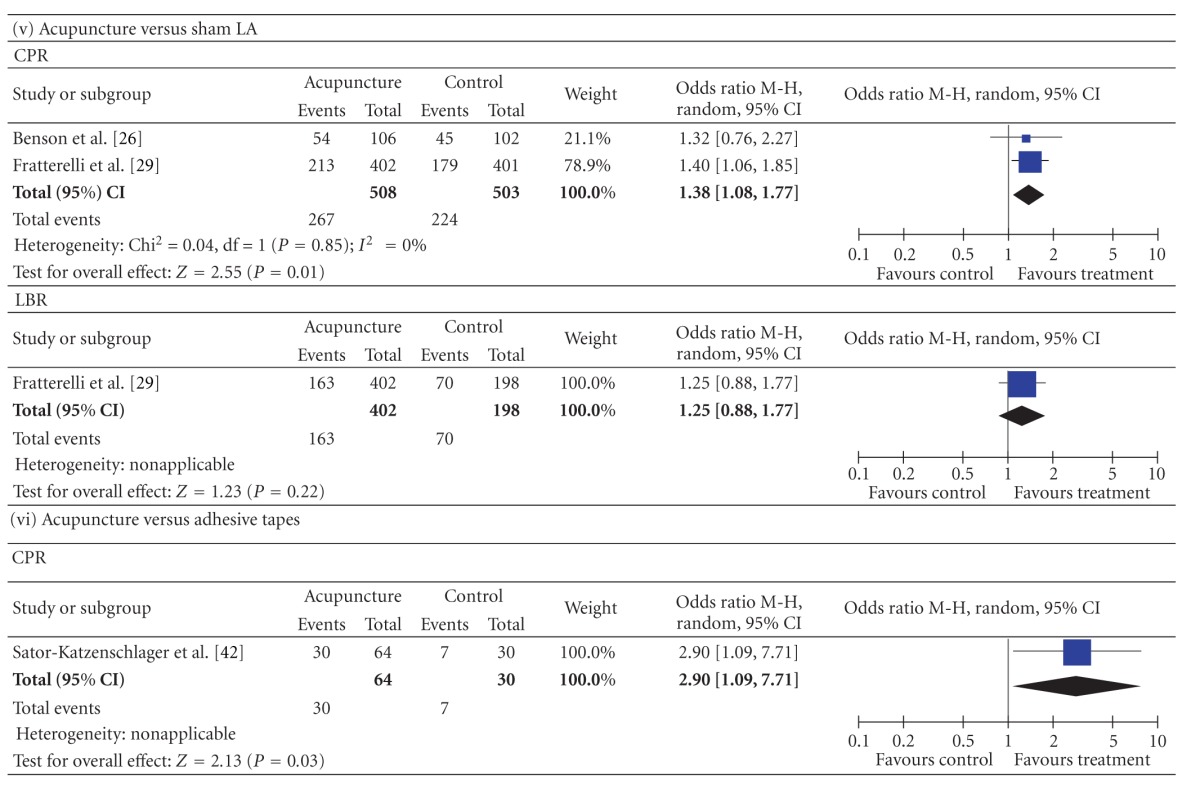

**Table 3:** Forest plots of IVF outcomes as compared by different acupuncture times and controls.

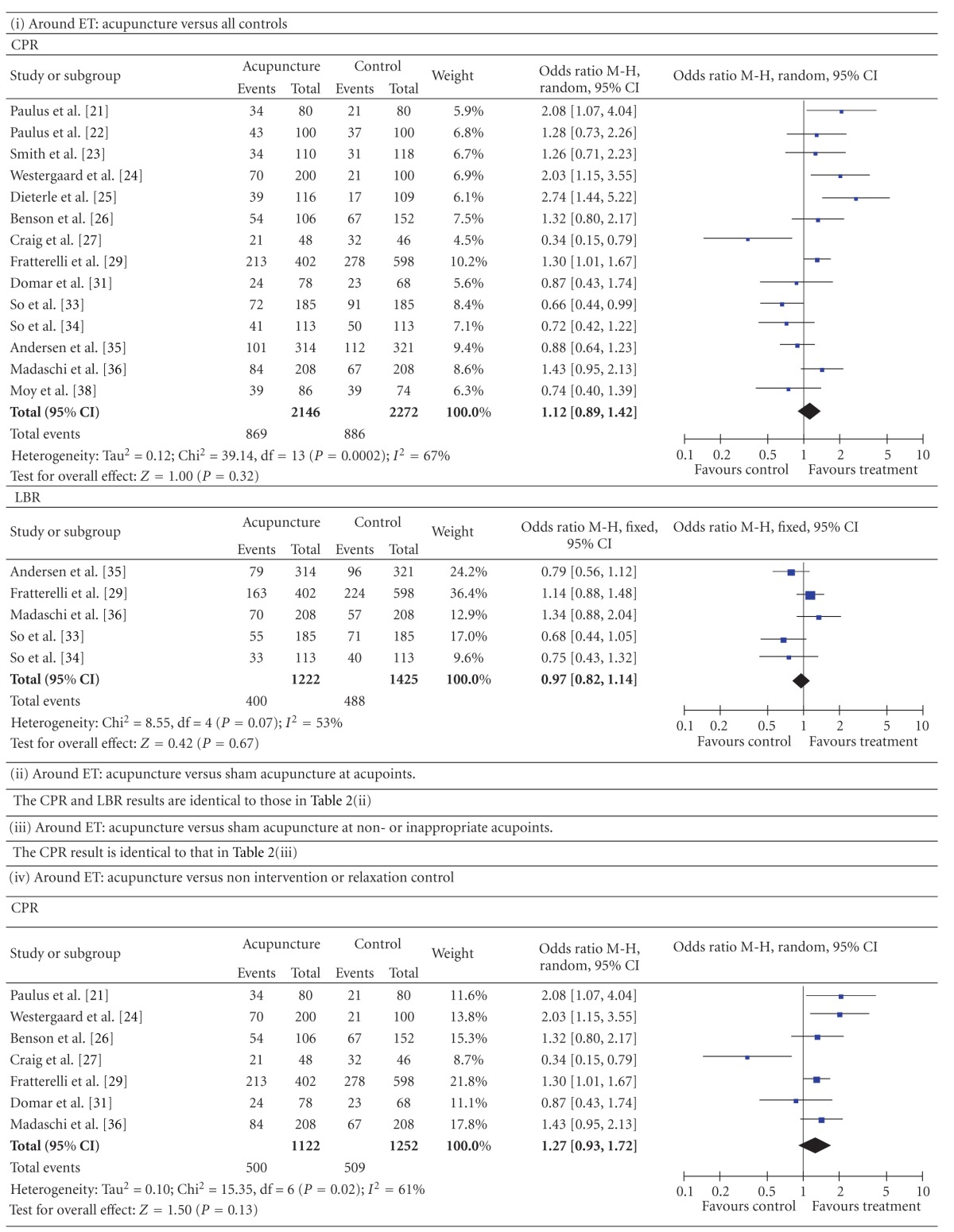 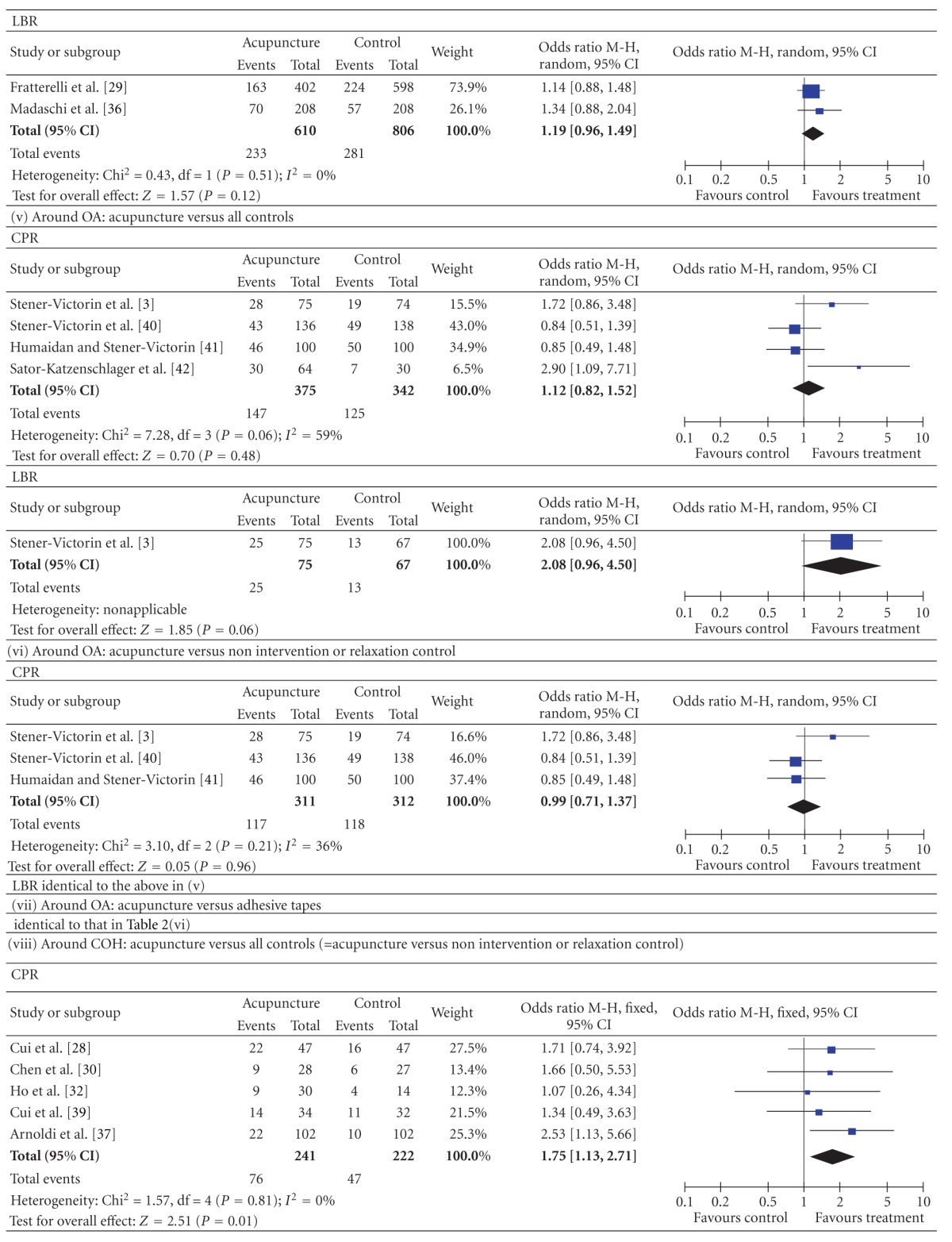

**Table 4:** Funnel plots of IVF outcomes as compared by different acupuncture times and controls.